# Gastrointestinal parasites of indigenous pigs (*Sus domesticus*) in south‐central Nepal

**DOI:** 10.1002/vms3.536

**Published:** 2021-05-22

**Authors:** Roshan B. Adhikari, Madhuri Adhikari Dhakal, Santosh Thapa, Tirth R. Ghimire

**Affiliations:** ^1^ Animal Research Laboratory Faculty of Science Nepal Academy of Science and Technology (NAST) Khumaltar, Lalitpur Nepal; ^2^ Third Pole Conservancy Bhaktapur Nepal; ^3^ Faculty of Science and Engineering Macquarie University Sydney NSW Australia; ^4^ Department of Pathology and Immunology Baylor College of Medicine Houston TX USA; ^5^ Texas Children's Microbiome Center Department of Pathology Texas Children's Hospital Houston TX USA; ^6^ Department of Zoology Tri‐Chandra Multiple Campus Tribhuvan University Kathmandu Nepal

**Keywords:** *Balantidium*, Chwanche, gastrointestinal parasites, Nepal, swine, zoonosis

## Abstract

**Background:**

Intestinal parasites have a significant impact on productivity of pigs. Additionally, presence of zoonotic parasites in pig faeces used as fertilizer and ingestion of raw or undercooked pork products originated from parasite‐infested pigs pose a risk to human health.

**Objectives:**

The aim of the study was to estimate the prevalence and diversity of gastrointestinal (GI) parasites in indigenous pigs (*Sus domesticus*) maintained under traditional rearing system in Nepal.

**Methods:**

Fresh faecal samples (*n* = 100) were collected from the pigs of varying age and sex maintained in 18 small‐scale farms in south‐central Nepal. Samples were processed using various standard methods and examined for parasite eggs, cysts or oocysts.

**Results:**

Prevalence of GI parasites in indigenous pigs was 91%, comprising of 14 different genera of protozoans and helminths. Male pigs generally had a higher (97.5%) prevalence of GI parasites than females (87%). While 90% of the suckling and weaner piglets were positive for the GI parasites, all growers and 85% the adult pigs were infected with the parasites. *Entamoeba* spp. were the primary protozoans in all age groups. *Strongyloides* sp. was more prevalent helminths in suckling and weaner piglets, whereas Ascarid spp. were higher in both growers and adults. Triplet infection was higher (33.3%) in suckling and weaner piglets, while quadruplet and pentuplet infections were higher (*p* < .05) among growers (46.7%) and adults (30%), respectively.

**Conclusions:**

The indigenous pigs harbour a higher prevalence and greater diversity of GI parasites. GI parasitism varies by sex and age of the pigs.

## Graphical abstract

The aim of the study was to estimate the prevalence and diversity of gastrointestinal (GI) parasites in indigenous pigs (*Sus domesticus*) maintained under traditional rearing system in Nepal. Faecal samples contained 14 different protozoa and helminths with the prevalence rates of 91%. The indigenous pigs harbour a higher prevalence and greater diversity of GI parasites.

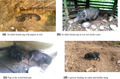

## 1 INTRODUCTION

Pigs are small, adaptable, rapidly growing and multiparous livestock species reared globally for their meat value. Their manure can also be used to produce biogas and as a soil fertilizer. For a long time, pig husbandry practices were restricted to very few ethnic communities in Nepal due to religious constraints and misconceptions of pork meat (Nidup et al., 2010). However, with increased urbanization, and cross‐culture experiences, people's perception regarding pig rearing and pork consumption has changed in recent years. That might be why the annual demand of pork in Nepal was reported to increase by 10% (Thapa, 2018), and the pork was in the 4th position of all the meat consumed across the country (Pant, 2017).

The Nepalese pig industry is relatively small, as represented by its number 13,55,659 in the first 8 months of the fiscal year 2018/2019 (GoN, 2019). The native or indigenous pig breeds of rural Nepal are Chwanche, Hurrah, Bampudke, Pakhribas Black and Dharane Kalo Bangur, and others (Anonymous, Unknown). It is usually common in Nepal to raise the indigenous pigs under traditional farm management (Paudel et al., 2014). Their feed mainly includes kitchen waste, garbage, roots, green forages and locally available grains like rice‐bran, maize, husks, and others (Nidup et al. 2010). In contrast, exotic breeds of pigs have been imported in Nepal since 1957 AD to upgrade the native pigs via crossbreeding. They include Tamworth, Saddleback, Fauyen, Landrace, Hampshire, Duroc and The Large White (Yorkshire White; Anonymous, Unknown) and are raised primarily by urban and commercial farmers with special care and feeds (Paudel et al., 2014). Whatever the farming breed and management practices adopted, the pork industry is believed to play a substantial role in meeting the food security and boosting the economy of Nepalese farmers.

While pig farming in Nepal supports the sustainable livelihood of farmers, it is still facing many challenges regarding productivity, profitability and sustainability. Some of the existing challenges are lack of sufficient meat‐processing factories and clean slaughterhouses, high feeding cost, poor market linkage, lack of adequate breeding farms, presence of primitive breeds (genetic threats), primitive model of farming practices and parasitic diseases (Gatenby & Chemjong, 1992; Paudel et al., 2014; Nidup et al., 2010). Among parasitic diseases, the gastrointestinal (GI) parasites are responsible for a substantial loss on the efficiency of pig production by competing directly for nutrients required for optimum growth and reproduction or by causing tissue injuries (lesions), leading to condemnation of organs during meat inspection, poor feed conversion, diarrhoea and dehydration or even death of the animals.

The prevalence of enteric parasites in pigs is widely reported across the globe. Some protozoan parasites, such as *Entamoeba* spp. (Matsubayashi et al., 2014, 2015), *Giardia* (Armson et al., 2009; Laber et al., 2002), *Eimeria* (Henry & Tokach, 1995; Jones et al., 1985), *Cystoisospora* (Chae et al., 1998; Johnson et al., 2008), *Cryptosporidium* (Argenzio et al., 1990, 1997), *Balantidium coli* (Laber et al., 2002; Sangioni et al., 2017) and helminth parasites such as *Fasciola* sp. (Capucchio et al., 2009; Horchner & Dalchow, 1972), *Trichuris* sp. (Batte et al., 1977; Laber et al., 2002), hookworm (Seguel & Gottdenker, 2017; Steenhard et al., 2000), strongyles (Patra et al., 2013; Sarashina & Taniyama, 1986), *Stongyloides* spp. (Giese et al., 1973; Laber et al., 2002), Ascarid spp. (Midttun et al., 2018; Ondrejková et al., 2012) are found to be associated with severe morbidity and mortality in pigs. *Ascaris suum*, *Strongyloides*, *Trichuris suis* and Strongyle have previously been reported from pigs in Nepal (Baskota & Shrestha, 2019; Sah, 2018). However, these reports included very few parasites and have not elaborated on how they could occur in pigs. Therefore, the objective of the study was to determine the prevalence and diversity of GI parasites of domestic pigs in Nepal. The resulting data on GI parasites in the pigs may aid in establishing effective and sustainable interventions to improve pig's health and the pig industries in Nepal.

## 2 MATERIALS AND METHODS

### 2.1 Study area

**FIGURE 1:**
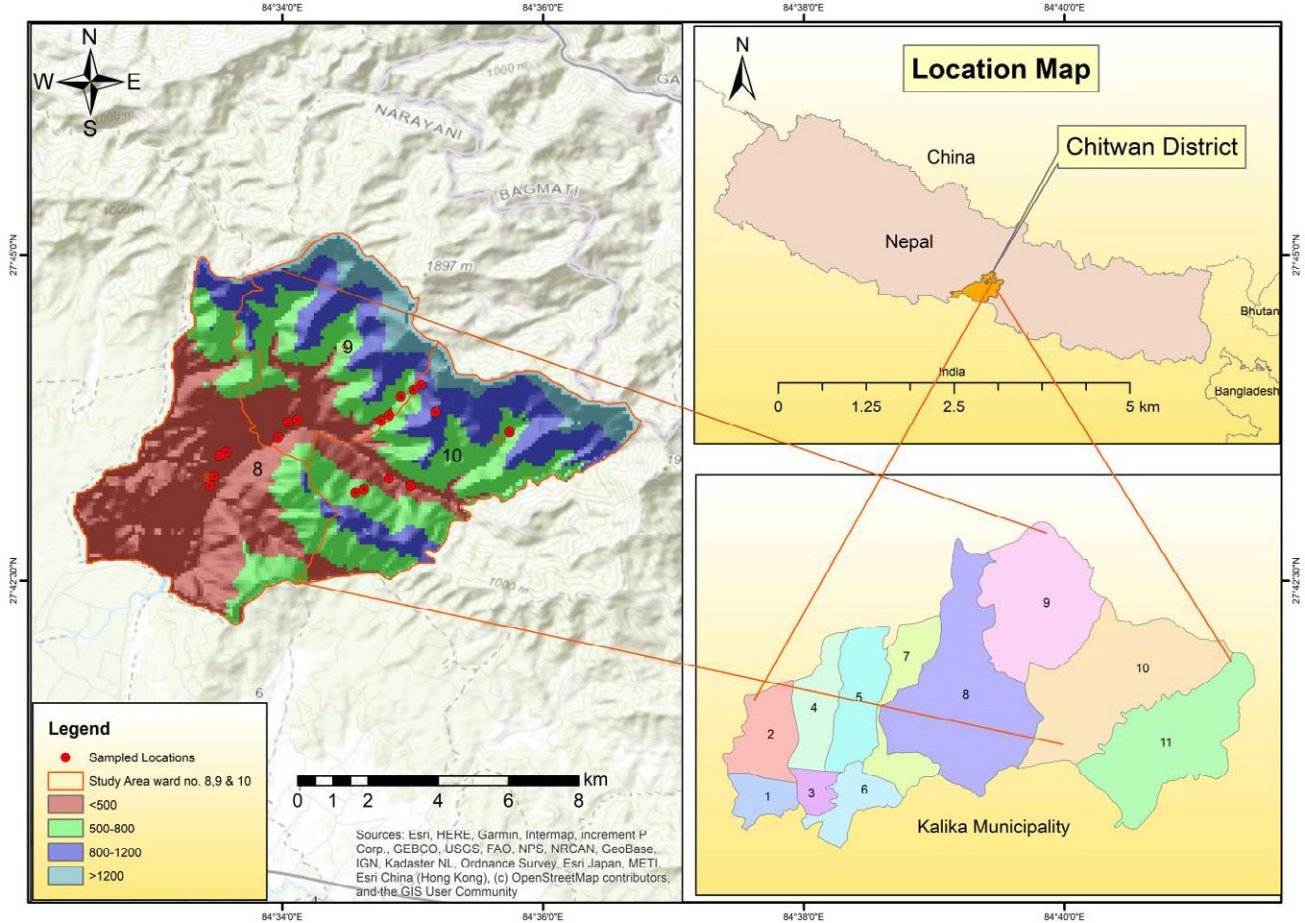
Map of the study area showing the locations of sample collection

**FIGURE 2:**
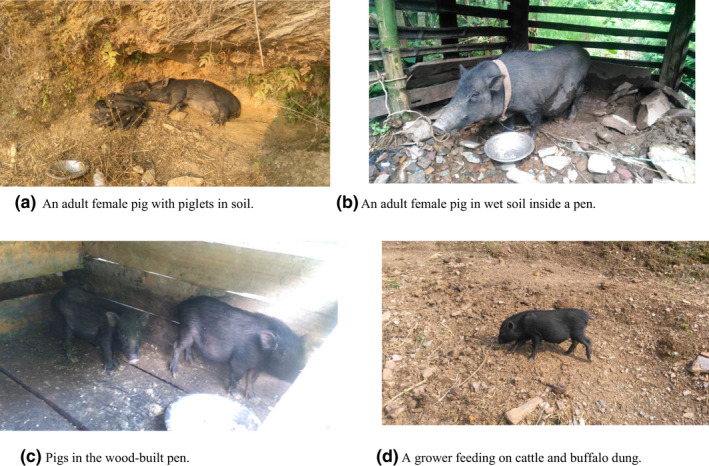
Pigs in different conditions. (a) An adult female pig with piglets in soil. (b) An adult female pig in wet soil inside a pen. (c) Pigs in the wood‐built pen. (d) A grower feeding on cattle and buffalo dung

The study was conducted from June to September 2019 in Shaktikhor area of Kalika Municipality (251–1,003 m above sea level, a.s.l.) in the Chitwan district of Nepal (Figure 1). The area lies in the southcentral part of Nepal and is approximately 182 km away from the capital city Kathmandu. The region experiences a tropical to subtropical climate with an annual mean temperature of 29.3°C in summer and 9.4°C in winter. The average yearly precipitation in the area is 199 mm (Adhikari et al., 2020). The indigenous/ethnic people living in the study area prefer traditional small‐scale pig rearing. Most of the household rears two to ten locally available indigenous breed *Sus domesticus* (“Chwanche” in the Nepali language; Figure 2) under scavenging management (Pant, 2017) at the backyard of their house (Associate Professor Dr. Nirajan Bhattarai, Department of Animal Breeding and Biotechnology, Faculty of Animal Science, Veterinary Science and Fisheries, Agriculture and Forestry University, Rampur, Chitwan, Nepal: personal communications). Pigs were categorized into suckling and weaner (piglets <4 months), growers (4–8 months), and adults (>8 months) groups as previously described (Sharma et al., 2020). The inclusion criteria of the survey farms were accessibility of the farms and voluntary participation of the pig farmers.

### 2.2 Sample collection

Approximately 10 g of fresh faecal samples were non‐invasively collected from 100 pigs (one sample per animal) of different ages and sexes owned by 18 smallholder farmers. Briefly, the topper layer of the stool that has not touched the ground immediately after defecation was collected with gloved hands. Utmost care was taken to avoid contamination of the samples. The samples were then placed into 20 ml screw‐cap sterile vials containing 2.5% weight/volume (w/v) potassium dichromate. To avoid duplicating the samples, the sampled pigs were marked with identifiers. The samples were immediately transported to the Animal Research Laboratory (ARL) of the Nepal Academy of Science and Technology (NAST) for analysis. Upon receipt at the ARL, the samples were stored at 4°C until further processing.

### 2.3 Sample processing and analysis

Each stool sample was processed using multiple methods: direct wet mount, sedimentation, saturated salt (45% w/v NaCl) flotation and acid‐fast techniques (Adhikari et al., 2021; Adhikari et al., 2020; Ghimire & Bhattarai, 2019). Samples positive for *Eimeria* sp. or *Cystoisospora* sp. were proceeded for the sporulation assays (Adhikari et al., 2020; Chhabra & Mafukidze, 1992). Samples were then examined for parasitic bodies such as cysts, trophozoites, oocysts and ova under a B‐383PLi light microscope (OPTIKA Microscopes) at 100×, 400× and 1,000× magnifications. Images of the parasites/parasitic bodies were taken by a camera (SXView 2.2.0.172 Beta (Nov 6, 2014)) attached to the microscope. The micrometry of parasites was calculated using ImageJ 1.51k (National Institute of Health). Identification of the parasites was made as previously described (Chhabra & Mafukidze, 1992; Soulsby, 2012; Widisuputri et al., 2020).

### 2.4 Parasite severity

Parasite severity was measured by quantifying the number of eggs of nematodes and oocysts of *Eimeria* and *Cystoisospora* released per gram of faeces (epg/opg) by applying the McMaster technique (Adhikari & Ghimire, 2021; Soulsby, 2012). We used the 2 Cell McMaster Counting Slide (Hawksley and Sons Ltd.) following the manufacturer's recommendations. Briefly, three grams each of coccidian and nematode‐positive stool samples were weighed and filtered through a tea strainer into a 50 ml beaker using 43 ml of floatation fluid made up of 45% NaCl. Then, 0.15 ml of the filtrate was placed into each depth of the McMaster slide using a pipette, and the slide was examined using a 100× total magnification under a compound microscope. All oocysts or eggs in both chambers were counted, and their sum was multiplied by 100. Finally, the resulting product was divided by 2 to calculate the epg or opg.

### 2.5 Data analysis

Data were expressed as a number of parasite positive samples (frequency) and prevalence in terms of percentage using the Microsoft Excel 2016 for Windows (Microsoft Corporation). The GraphPad Prism Software Windows v5.00 (GraphPad Software, Inc.) was used to perform statistical analysis of the data. The chi‐square (*χ* ^2^) test was used to compare the differences in the prevalence of GI parasites by sex and age of the pigs. For all analyses, *p* < .05 (at 95% confidence interval) was considered statistically significant.

## 3 RESULTS

**FIGURE 3:**
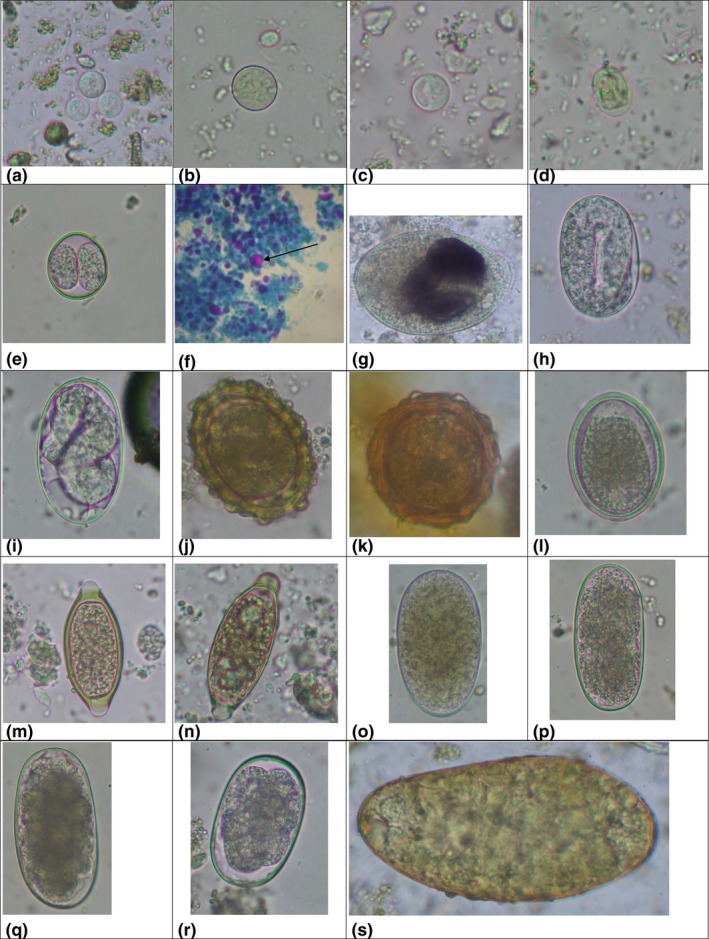
Gastrointestinal parasites identified in the pigs. (a) Cysts of *Entamoeba* spp. (9–11 × 9–10 µm), 400×, direct wet mount at Gram's iodine stain. (b) Cyst of *Entamoeba coli* (18 × 16 µm), 400×, after sedimentation technique at Gram's iodine stain. (c) Cyst of *Iodamoeba butschlii* (15 × 14 µm), 400×, after sedimentation technique. (d) Cyst of *Giardia* sp. (13 × 9 µm), 400×, direct wet mount at Gram's iodine stain. (e) Oocyst of *Cystoisospora* sp. (27 × 23 µm), 400×, after flotation technique. (f) Oocyst of *Cryptosporidium* sp. (4 × 4 µm), 1,000×, after acid‐fast staining (shown by arrow). (g) Trophozoite of *Balantidium coli* (98 × 61 µm), 400×, direct wet mount at Gram's iodine stain. (h) Egg of *Strongyloides* sp. (66 × 39 µm), 400×, after flotation technique. (i) Egg of *Strongyloides* sp. (51 × 33 µm), 400×, after sedimentation technique at Gram's iodine stain. (j) Egg of Ascarid sp. (1) (73 × 56 µm, 400×, after flotation technique). (k) Egg of Ascarid sp. (2) (56 × 56 µm, 400×, after flotation technique). (l) Decorticated egg of Ascarid sp. (60 × 44 µm, 400×, after flotation technique). (m) Egg of *Trichuris* sp. (1) (58 × 26 µm, 400×, after flotation technique). (n) Egg of *Trichuris* sp. (2) (67 × 24 µm), 400×, after sedimentation technique at Gram's iodine stain. (o). Egg of strongyle (1) (112 × 68 µm), 400×, after flotation technique. (p) Egg of strongyle (2) (89 × 44 µm), 400×, after flotation technique. (q) Egg of strongyle (3) (63 × 39 µm), 400×, after flotation technique. (r) Egg of hookworm (69 × 38 µm), 400×, after flotation technique). (s) Egg of *Fasciola* sp. (146 × 70 µm), 400×, after sedimentation technique at Gram's iodine stain

**FIGURE 4:**
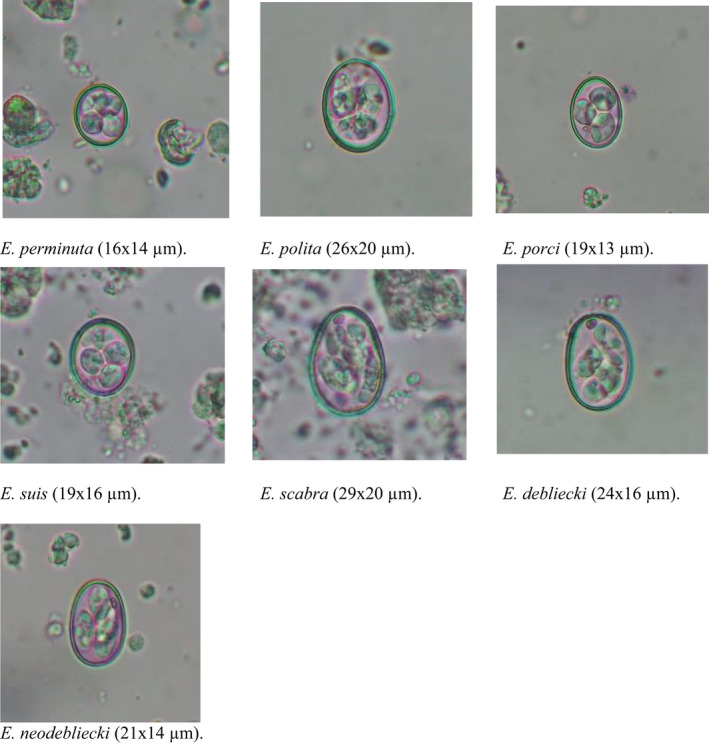
Different species of *Eimeria* in the faecal samples of pigs after flotation techniques (400×)

A total of 91 faecal samples (91%) had one or more GI parasites. Protozoan parasites were highly (89%) prevalent in the pigs than the helminths (75%; Table 1). Regarding protozoa, *B. coli*, *Cryptosporidium* sp., *Entamoeba coli*, *Entamoeba* spp., *Eimeria neodebliecki*, *E. debliecki*, *E. suis*, *E. perminuta*, *E. porci*, *E. polita*, *E*. *scabra*, *Giardia* sp., *Iodamoeba butschlii*, and *Cystoisospora* sp. were detected in the samples (see Table 1; Figures 3 and 4; Table S1). In contrast, helminths identified included Ascarid spp., *Fasciola* sp., hookworm, strongyle, *Strongyloides* sp. and *Trichuris* spp. The three morphotypes of eggs of Ascarid spp. (corticated oval, corticated round and decorticated smooth) were also detected. Similarly, ova of different *Trichuris* spp. (long and thin form and short and broad form; size: 56–67 µm × 24–30 µm) were recorded. Due to the lack of faecal culture and identification of third‐stage larvae for a complete diagnosis, we considered ''strongylid'' for the strongyle‐type eggs of *Oesophagostomum,* *Hyostrongylus*, *Globocephalus* and *Trichostrongylus* (Adhikari et al., 2020; Gatenby & Chemjong, 1992; Ghimire & Bhattarai, 2019). Strongylid eggs were of four types depending on size (70–135 µm × 34–77 µm) and shape (oval to rectangular; see Table 1; Figure 3, 4).

**TABLE 1:** Age‐wise prevalence of gastrointestinal parasites in domestic pigs of south‐central Nepal

Parasites	Sucklings and weaners	Growers	Adults	Overall	*p*‐values
Protozoa					
*Entamoeba* spp.	17 (56.7)	19 (63.3)	25 (62.5)	61 (61)	ns
*Eimeria* spp.	16 (53.3)	17 (56.7)	14 (35)	47 (47)	*p* < .05
*Balantidium coli*	4 (13.3)	9 (30)	15 (37.5)	28 (28)	*p* < .05
*Cystoisospora* sp.	7 (23.3)	6 (20)	8 (20)	21 (21)	ns
*Entamoeba coli*	1 (3.3)	5 (16.7)	5 (12.5)	11 (11)	*p* < .05
*Cryptosporidium* sp.	5 (16.7)	3 (10)	2 (5)	10 (10)	*p* < .05
*Idoamoeba butschlii*	0 (0)	2 (6.7)	6 (15)	8 (8)	*p* < .05
*Giardia* sp.	4 (13.3)	1 (3.3)	2 (5)	7 (7)	*p* < .05
Total	26 (86.7)	29 (96.7)	34 (85)	89 (89)	*p* < .05
Helminths					
Ascarid spp.	3 (10)	16 (53.3)	26 (65)	45 (45)	*p* < .05
Strongyle	0 (0)	14 (46.7)	18 (45)	32 (32)	*p* < .05
*Trichuris* spp.	5 (16.7)	12 (40)	13 (32.5)	30 (30)	*p* < .05
*Strongyloides* sp.	7 (23.3)	6 (20)	10 (25)	23 (23)	ns
Hookworm	3 (10)	4 (13.3)	13 (32.5)	20 (20)	*p* < .05
*Fasciola* sp.	0 (0)	5 (16.7)	4 (10)	9 (9)	*p* < .05
Total	14 (46.7)	29 (96.7)	32 (80)	75 (75)	*p* < .05
Grand total	27 (90)	30 (100)	34 (85)	91 (91)	*p* < .05

Note

Sucklings and weaners (*n* = 30), Growers (*n* = 30), Adults: (*n* = 40). Total numbers of samples = 100. Values in brackets indicate the % prevalence. *p*‐Values were assessed by comparing the prevalence rates of individual parasite among three age‐groups using Chi‐square tests.

Abbreviation: ns, not significant.

Regarding age‐wise distribution, we found a 90% prevalence of enteric parasites in suckling and weaner piglets, 100% in growers and 85% in adult pigs (Table 1). *Entamoeba* spp. were primary protozoa in all three age groups. Regarding helminths, *Strongyloides* sp. was higher in suckling and weaner piglets, whereas Ascarid spp. were higher in both growers and adult pigs.

The overall prevalence of GI parasites was higher in male pigs (95.7%) than the females (87%), without statistical significance (*p* >.05). The prevalence of enteric parasites (protozoans and helminths combined) among suckling and weaner piglets was higher in females than males. Growers had a similar prevalence of the parasites in both sexes, while the prevalence was higher in males among adult pigs (Table 2).

**TABLE 2:** Age‐ and sex‐wise distribution of polyparasitism in domestic pigs in south‐central Nepal

Number of parasitic infestations	Suckling and weaner		Growers		Adults		Overall		Grand total (*N* = 100)	*p* values
	Male (*n* = 18)	Female (*n* = 12)	Male (*n* = 14)	Female (*n* = 16)	Male (*n* = 14)	Female (*n* = 26)	Male (*n* = 46)	Female (*n* = 54)		
Single	4 (22.2)	2 (16.7)	0 (0)	0 (0)	0 (0)	0 (0)	4 (8.7)	2 (3.7)	6 (6)	*p* < .05
Duplet	3 (16.7)	1 (8.3)	1 (7.1)	2 (12.5)	2 (14.3)	0 (0)	6 (13)	3 (5.6)	9 (9)	
Triplet	6 (33.3)	4 (33.3)	2 (14.3)	4 (25)	1 (7.1)	2 (7.7)	9 (19.6)	10 (18.5)	19 (19)	
Quadruplet	3 (16.7)	4 (33.3)	6 (42.9)	8 (50)	4 (28.6)	5 (19.2)	13 (28.3)	17 (31.5)	30 (30)	
Pentuplet	0 (0)	0 (0)	3 (21.4)	0 (0)	5 (35.7)	7 (26.9)	8 (17.4)	7 (13)	15 (15)	
Hexuplet	0 (0)	0 (0)	2 (14.3)	2 (12.5)	1 (7.1)	3 (11.5)	3 (6.5)	5 (9.3)	8 (8)	
Septuplet	0 (0)	0 (0)	0 (0)	0 (0)	1 (7.1)	3 (11.5)	1 (2.2)	3 (5.6)	4 (4)	
Total	16 (88.9)	11 (91.7)	14 (100)	16 (100)	14 (100)	20 (76.9)	44 (95.7)	47 (87)	91 (91)	

Note

Suckling and Weaner (*n* = 30), Growers (*n* = 30), Adults (*n* = 40). *n* denotes the numbers of samples from male and female pigs. Data are expressed in the numbers of positive samples and prevalence (in brackets).

Regarding polyparasitism, triplet infection (33.3%) was higher among the suckling and weaner piglets, while quadruplet and pentuplet infections were higher among the growers (46.7%) and adults (30%), respectively. However, among all positive samples with polyparasitism, quadruplet infection (30%) was the highest, and septuplet infection (4%) was the lowest (Table 1) (Table S2). Of seven *Cystoisospora* and *Eimeria* positive samples, most samples (10%) were from adults, whereas 6.7% samples were from growers. These parasites were found in only one sample of sucklings and weaners (Table S3). Age‐ and sex‐wise distribution of polyparasitim in those pigs was statistically significant (*p* < .05; Table 2).

The range of opg of faeces for *Eimeria* spp. was highest (400–6,200) in suckling and weaner piglets, whereas opg for *Cystoisospora* sp. was highest in the growers (100–2,400). On the other hand, the epg count of Ascarid spp. (100–7,800), hookworm (100–1,600), *Strongyloides* sp. (100–2,200) and *Trichuris* spp. (100–1,500) was higher in adult pigs, and that of the strongyle (100–4,200) was higher in the growers (Table S4).

## 4 DISCUSSION

The present study investigated the prevalence and diversity of GI parasites in indigenous pigs of Nepal. Our findings of 91% overall prevalence of GI parasites in the pigs are in concordance with the findings from Burkina Faso and Uganda (91%; Nissen et al., 2010; Tamboura et al., 2006), but slightly lower than that of the reports from Indonesia (100%; Widisuputri et al., 2020), Bangladesh (96.4%; Dey et al., 2014), Brazil (93.1%; Barbosa et al., 2015). In contrast, our result was higher than the reports from Kenya (83%–84.2%; Kagira et al., 2012; Obonyo et al., 2013), Tanzania (83%; Nonga & Paulo, 2015), South Africa (79.2%; Nwafor et al., 2019) and Korea (73.5%; Ismail et al., 2010). The difference in the prevalence of parasites in these studies can be attributed to many factors such as age, sex and breeds of the pigs and their immune system, the diversity in the climate or sampling season, landscapes of sampling sites and husbandry practices, variation in sample size and the laboratory techniques for faecal analysis.

One of the reasons for the higher prevalence of GI parasites in this study could be due to the poor rearing condition of the pigs. Many farmers in the study area were unaware of effective pig‐rearing and farm management practices. In Nepal, pig farmers usually do not pay much attention to the indigenous breeds compared with the crossbreds (Gatenby & Chemjong, 1992). The sampled indigenous pigs were fed inadequately and untimely and kept in wood‐built and untidy pens with the porous floor (Figure 2). These components generate higher moisture and attract mechanical vectors such as flies in the pens that may have contributed to the acquisition of diverse parasites.

The methodological variation could be another potential contributor to the higher prevalence of enteric parasites in the study. We have applied multiple techniques for faecal analysis, including direct wet mount, floatation, sedimentation, acid‐fast staining and sporulation, which might have cumulatively contributed to higher detection rates of the enteric parasites. More importantly, pigs themselves are the natural reservoir of many GI parasites recorded in this study (Ji et al., 2019; Schuster & Ramirez‐Avila, 2008). Indigenous breeds also naturally possess a high GI parasitic rate (Murthy et al., 2016), possibly contributing to a high prevalence rate in the faecal samples studied.

We found the highest parasitic prevalence (100%) in growers and the lowest (85%) in adult pigs. Similar results were also reported from Tanzania (Nonga & Paulo, 2015) and India (Sharma et al., 2020). The higher prevalence of enteric parasites in the growers might be due to their higher exposure to the outside environment. After completing the weaning period, they need to search for food themselves; thus, they forage on grasses in the open fields and get exposed to various parasites. On the other hand, the lower prevalence of GI parasites in adult pigs could be due to the enhanced resistance and susceptibility to reinfection governed by an increased immunological memory (Brake, 2003).

The findings of a higher prevalence of GI parasites in males lie in agreement with other published reports (Dey et al., 2014; Sharma et al., 2020; Sowemimo et al., 2012). The lower GI parasitism in females in our study could be due to deworming practice performed by few farmers (field observation) for adult pregnant pigs in pre‐farrowing condition (2 weeks before farrowing). Additionally, testosterone hormone, which acts as an immunosuppressant (Salvador et al., 1996), could have contributed to the higher prevalence of GI parasites in male pigs. However, further study is required to test this hypothesis.

Regarding the diversity of parasites, *Entamoeba* spp. had the highest prevalence rate (61%). This rate was lower than the findings from Indonesia (99%; Widisuputri et al., 2020), Kenya (87%) (Kagira et al., 2012), but higher than that reported from China (55.4%; Ji et al., 2019), United Kingdom (52.4%; Jacob et al., 2016) and Brazil (18.6%–44.3%; Barbosa et al., 2015). Various amoeba species have been previously reported in pigs, such as *E. suis*, *E. histolytica* and *E. polecki* (Ji et al., 2019; Mendoza‐Gómez et al., 2015). In addition to the pathogenic species, non‐pathogenic amoeba like *E*. *coli* and *I. butschlii* were also reported in the current study. Compared with the studies from Spain (44%; Bornay‐Llinares et al., 2006) and Colombia (40%; Mendoza‐Gómez et al., 2015), we found a lower prevalence of *E. coli* (11%) in the pigs, but higher than the results from Nigeria (2%; Ejinaka & Onyali, 2020). Similarly, the prevalence of *I. butschlii* (8%) was lower than the findings from Colombia (7.5%–57%; Mendoza‐Gómez et al., 2015) and Italy (25%; Cacciò et al., 2012). These data indicate the global distribution of the amoeba in pigs.

In terms of coccidia, we found *Eimeria*, *Cystoisospora* and *Cryptosporidium* species in the pigs studied. Our findings of 47% prevalence of *Eimeria* spp. was lower than the findings from Indonesia (78%; Widisuputri et al., 2020) and Bangladesh (56.4%; Dey et al., 2014) but higher than those reported from Japan (40.3%; Matsubayashi et al., 2009), Kenya (5%–40%; Kagira et al., 2012), India (24.63%; Patra et al., 2019) and China (16.53%; Lai et al., 2011). Notably, the present study firstly reported the seven species of *Eimeria* in pigs from Nepal, the total known *Eimeria* species being eight (Chhabra & Mafukidze, 1992; Sharma et al., 2020). Similarly, the prevalence of *Cystoisospora* sp. (21%) in the current study was lower than that reported from Germany (53.8%; Meyer et al., 1999) and Netherlands (53%; Eysker et al., 1994), but higher than those from Korea (17.3%; Chae et al., 1998), India (12.40%; Patra et al., 2019) and Bangladesh (9.1%; Dey et al., 2014). On the other hand, 10% prevalence of *Cryptosporidium* sp. in this study was in accordance with a study from India (10.66%; Patra et al., 2019), but lower than those reported from Canada (66.4%; Farzan et al., 2011), Ireland (44.6%; Xiao et al., 2006) and Denmark (40.9%; Petersen et al., 2015), and slightly higher than those from Turkey (8.8%; Uysal et al., 2009) and USA (8.6%; Xiao et al., 1994). Several species of *Cryptosporidium* such as *C. suis*, *C. muris*, *C. parvum* (animal genotype) and *C. scrofarum* (*C*. Pig genotype II), reported in previous studies of pigs (Kváč et al., 2009, 2013; Xiao et al., 2006) suggest the importance of further molecular studies of these parasites in pigs from Nepal.

*Giardia* sp. was the only flagellate found in 7% of our samples. This rate was similar to the findings from USA (7.4%; Kváč et al., 2009), but lower than that reported from Canada (66.4%; Farzan et al., 2011), the United Kingdom (57.1%; Minetti et al., 2014), Australia (31.1%; Armson et al., 2009) and Denmark (14%; Petersen et al., 2015), and higher than reported from India (7.63%; Patra et al., 2019) and Turkey (3.7%; Uysal et al., 2009).

Interestingly, *B. coli*, a common natural parasite of pigs, was reported in 28% of the samples. This rate was lower than the findings from Indonesia (79%; Widisuputri et al., 2020), Brazil (46.4%–71.6%; Barbosa et al., 2015), Korea (64.7%; Ismail et al., 2010), Kenya (64%; Kagira et al., 2012), Colombia (42%; Mendoza‐Gómez et al., 2015), Bangladesh (40%; Dey et al., 2014) and India (29.48%; Patra et al., 2019) but higher than those reported from China (22.79%; Lai et al., 2011), Malaysia (22%; Tan et al., 2014) and Nigeria (13%; Bernard et al., 2015).

Regarding helminths, Ascarid spp. were reported in 45% of the examined samples. This prevalence rate was lower than the findings from Netherlands (72.7%; Eijck & Borgsteede, 2005), Botswana (54.55%; Nsoso et al., 2000), Bangladesh (50.9%; Dey et al., 2014) and South Africa (44.5%; Nwafor et al., 2019) and higher than those from Burkina Faso (40%; Tamboura et al., 2006), Uganda (40%; Nissen et al., 2010), Tanzania (37%; Nonga & Paulo, 2015) and India (33.3%; Patra et al., 2019). Strongyle was the second most prevalent nematode. The 32% prevalence report of this parasite was lower than the findings from Uganda (89%; Nissen et al., 2010), Kenya (75%; Obonyo et al., 2013), Tanzania (52%; Nonga & Paulo, 2015) and Brazil (46.6%; Barbosa et al., 2015) but higher than the findings from Ghana (11%; Atawalna et al., 2016) and India (11.10%; Patra et al., 2019). *Trichuris* spp. were recorded in the 30% samples. This rate was lower than the findings from Kenya (78%; Obonyo et al., 2013), South Africa (50.6%; Nwafor et al., 2019) and Netherlands (37.5%; Eijck & Borgsteede, 2005) and higher than the results from India (17.3%–27.84%; Murthy et al., 2016; Patra et al., 2019), Japan (24.8%; Matsubayashi et al., 2009), Indonesia (20%; Widisuputri et al., 2020) and Uganda (17%; Nissen et al., 2010). Similarly, the 23% prevalence rate of *Strongyloides* sp. was lower than the findings from Bangladesh (29.1%; Dey et al., 2014) and Kenya (26.6%; Obonyo et al., 2013) and higher than the results from Burkina Faso (21%; Tamboura et al., 2006), Indonesia (19%; Widisuputri et al., 2020), Tanzania (15%; Nonga & Paulo, 2015) and India (12.74%; Petersen et al., 2015). Hookworm was the least reported nematode, detected in only 20% of the samples. However, the rate was higher than the findings from Nigeria (3.5%–5.9%; Ejinaka & Onyali, 2020; Sowemimo et al., 2012) and Bangladesh (3.6%; Dey et al., 2014).

Interestingly, *Fasciola* sp. was the only trematode reported in this study. Its prevalence rate (9%) was similar to the findings from Nigeria (9.3%; Bernard et al., 2015), but lower than those reported from Bolivia (27.1%; Mas‐Coma et al., 1997) and Chile (20.6%; Apt et al., 1993), and higher than those from Italy (4.37%; Capucchio et al., 2009) and China (1.3%; Boes et al., 2000). Adult pigs possess a greater natural resistance to the infection because the fibrous nature of their liver parenchyma and matured immune response act as mechanical barriers for the migrating metacercaria (Nansen et al., 1972; Ross et al., 1967). However, the newly born pigs can acquire *Fasciola* infections more readily than adults (Nansen et al., 1972). These hosts can also act as a secondary reservoir for this trematode and possess transmission potentiality (Mas‐Coma et al., 1997).

We also assessed the polyparasitism of the GI parasites in pigs. The current rate of 85% concurrency and the maximum number of faecal samples with triplet to pentuplet infections indicate dominant polyparasitism and high intensity of endoparasites in the pigs. Multi‐parasitism is associated with greater exploitation of the host defense mechanism (Schjørring & Koella, 2003) and may impact negatively (Vaumourin et al., 2015). For example, mixed intestinal infections, including *Cryptosporidium* sp., led to piglet death in Australia (Morgan et al., 1999). Similarly, cystoisosporiasis with any other parasitic or bacterial or viral infections leads to excessive mortality (Worliczek et al., 2007). The parasitic severity measured by epg or opg suggests that pigs were severely infected with various species of *Eimeria*, *Cystoisospora*, *Ascaris*, strongyle, *Strongyloides*, hookworm and *Trichuris*. Thus, the effects of mixed infections by all pathogen communities, rather than a single species, should be evaluated while assessing the severity of infections and the resulting pathologies (Serrano & Millán, 2014; Zhang et al., 2016).

## 5 CONCLUSIONS

The indigenous pigs (*S. domesticus*) maintained under traditional rearing system in Nepal had a higher prevalence (91%) GI parasites (protozoans and helminths). GI parasitism varied by sex and age of the pigs. Our findings suggest that indigenous pigs under traditional management can harbour a wide variety of GI parasites with higher prevalence. Thus, periodic trainings on practices for healthy and sustainable pig husbandry should be conducted targeting rural pig farmers. Additionally, deworming practices could help them to achieve maximum productivity and reduce the risk of transmitting potential pig‐borne zoonotic diseases.

## CONFLICT OF INTEREST

The authors declare that there are no conflicts of interest.

## AUTHOR CONTRIBUTION

**Roshan Babu Adhikari:** Conceptualization; Formal analysis; Investigation; Writing‐original draft. **Madhuri Adhikari Dhakal:** Writing‐review & editing. **Santosh Thapa:** Validation; Writing‐review & editing. **Tirth Raj Ghimire:** Conceptualization; Formal analysis; Methodology; Project administration; Resources; Supervision; Writing‐review & editing.

## ETHICS APPROVAL

The authors have adhered to ethical policies of the journal. We declare that the study was conducted on the faecal samples of naturally infected pigs and no experimental infection of the pigs was performed during the study. The fieldwork was conducted with permission from the Kalika Municipality (Chitwan, Nepal) and the Kalika Municipality Veterinary Services (Chitwan, Nepal) (Permission no. 07/2075/76).

## Supporting information

Table S1

Table S2

Table S3

Table S4

## Data Availability

All data generated or analysed during this study are included in the main text of the manuscript and the [Link], [Link], [Link], [Link] files.

## References

[vms3536-bib-0001] Adhikari, J. N., Adhikari, R. B., Bhattarai, B. P., Thapa, T. B., & Ghimire, T. R. (2021). A small‐scale coprological survey of the endoparasites in the Himalayan goral *Naemorhedus goral* (Hardwick, 1825) in Nepal. Biodiversitas Journal of Biological Diversity, 22. 10.13057/biodiv/d220326

[vms3536-bib-0002] Adhikari, R. B., & Ghimire, T. R. (2021). A Case study of multiple parasitisms in a calf buffalo (*Bubalus bubalis*). Agricultural Science Digest, 41, 237–241. 10.18805/ag.D-5172

[vms3536-bib-0003] Adhikari, R. B., Maharjan, M., & Ghimire, T. R. (2020). Prevalence of gastrointestinal parasites in the frugivorous and the insectivorous bats in Southcentral Nepal. Journal of Parasitology Research, 2020. 10.1155/2020/8880033 PMC7752302 33414955

[vms3536-bib-0004] Anonymous . (Unknown). Pig farm [Online]. Organic farm Nepal (Agriculture in Nepal), Himalaya Organic Farm Nepal Pvt. Ltd. Available from http://www.agricultureinnepal.com/pig‐farm. Accessed April 26, 2021

[vms3536-bib-0005] Apt, W., Aguilera, X., Vega, F., Alcaíno, H., Zulantay, I., Apt, P., González, V., Retamal, C., Rodríguez, J., & Sandoval, J. (1993). Prevalence of fascioliasis in humans, horses, pigs, and wild rabbits in 3 Chilean provinces. Boletin De La Oficina Sanitaria Panamericana Pan American Sanitary Bureau, 115, 405–414. 8274227

[vms3536-bib-0006] Argenzio, R. A., Armstrong, M., & Rhoads, J. M. (1997). Role of the enteric nervous system in piglet cryptosporidiosis. Journal of Pharmacology and Experimental Therapeutics, 279, 1109–1115. 8968331

[vms3536-bib-0007] Argenzio, R., Liacos, J., Levy, M., Meuten, D., Lecce, J., & Powell, D. (1990). Villous atrophy, crypt hyperplasia, cellular infiltration, and impaired glucose‐Na absorption in enteric cryptosporidiosis of pigs. Gastroenterology, 98, 1129–1140. 10.1016/0016-5085(90)90325-u 2323506

[vms3536-bib-0008] Armson, A., Yang, R., Thompson, J., Johnson, J., Reid, S., & Ryan, U. M. (2009). *Giardia* genotypes in pigs in Western Australia: Prevalence and association with diarrhea. Experimental Parasitology, 121, 381–383. 10.1016/j.exppara.2009.01.008 19271283

[vms3536-bib-0009] Atawalna, J., Attoh‐Kotoku, V., Folitse, R., & Amenakpor, C. (2016). Prevalence of gastrointestinal parasites among pigs in the Ejisu Municipality of Ghana. Scholars Journal of Agriculture and Veterinary Sciences, 3, 33–36.

[vms3536-bib-0010] Barbosa, A. S., Bastos, O. M., Dib, L. V., Siqueira, M. P. D., Cardozo, M. L., Ferreira, L. C., Chaves, W. T., Fonseca, A. B. M., Uchôa, C., & Amendoeira, M. R. R. (2015). Gastrointestinal parasites of swine raised in different management systems in the State of Rio de Janeiro, Brazil. Pesquisa Veterinaria Brasileira, 35, 941–946. 10.1590/S0100-736X2015001200001

[vms3536-bib-0011] Baskota, N., & Shrestha, S. (2019). Helminth parasites of pigs and development of suitable strategy for its control. Nepalese Veterinary Journal, 36, 163–169. 10.3126/nvj.v36i0.27776

[vms3536-bib-0012] Batte, E., Mclamb, R., Muse, K., Tally, S., & Vestal, T. (1977). Pathophysiology of swine trichuriasis. American Journal of Veterinary Research, 38, 1075–1079. 883715

[vms3536-bib-0013] Bernard, A. N., Daminabo, V., Ekam, E., Okonkwo, E., Nwuzo, A., Afiukwa, F., & Agah, M. (2015). Prevalence of intestinal parasites in faecal droppings of swine in Pankshin urban, Pankshin local government area, Plateau state, Nigeria. American Journal of Life Sciences, 3, 119–122. 10.11648/j.ajls.20150302.19

[vms3536-bib-0014] Boes, J., Willingham, A., Fuhui, S., Xuguang, H., Eriksen, L., Nansen, P., & Stewart, T. (2000). Prevalence and distribution of pig helminths in the Dongting Lake Region (Hunan Province) of the People's Republic of China. Journal of Helminthology, 74, 45–52. 10.1017/S0022149X00000068 10831052

[vms3536-bib-0015] Bornay‐Llinares, F. J., Navarro‐I‐Martínez, L., García‐Orenes, F., Araez, H., Pérez‐Murcia, M. D., & Moral, R. (2006). Detection of intestinal parasites in pig slurry: A preliminary study from five farms in Spain. Livestock Science, 102, 237–242. 10.1016/j.livsci.2006.03.023

[vms3536-bib-0016] Brake, D. A. (2003). Parasites and immune responses: Memory illusion? DNA and Cell Biology, 22, 405–419. 10.1089/104454903767650676 12906734

[vms3536-bib-0017] Cacciò, S. M., Sannella, A. R., Manuali, E., Tosini, F., Sensi, M., Crotti, D., & Pozio, E. (2012). Pigs as natural hosts of *Dientamoeba fragilis* genotypes found in humans. Emerging Infectious Diseases, 18, 838–841. 10.3201/eid1805.111093 22515838 PMC3358053

[vms3536-bib-0018] Capucchio, M. T., Deborah, C., Miriam, R., Vincenzo, A., Amedeo, T., Alessandro, L., Stefano, A., Eleonora, S. F., Bruno, D., & Franco, G. (2009). Natural trematode infestation in feral Nebrodi Black pigs: Pathological investigations. Veterinary Parasitology, 159, 37–42. 10.1016/j.vetpar.2008.10.017 19038498

[vms3536-bib-0019] Chae, C., Kwon, D., Kim, O., Min, K., Cheon, D., Choi, C., Kim, B., & Suh, J. (1998). Diarrhoea in nursing piglets associated with coccidiosis: Prevalence, microscopic lesions and coexisting microorganisms. Veterinary Record, 143, 417–420. 10.1136/vr.143.15.417 9807791

[vms3536-bib-0020] Chhabra, R., & Mafukidze, R. (1992). Prevalence of coccidia in pigs in Zimbabwe. Veterinary Parasitology, 41, 1–5. 10.1016/0304-4017(92)90002-q 1561754

[vms3536-bib-0021] Dey, T. R., Dey, A. R., Begum, N., Akther, S., & Barmon, B. C. (2014). Prevalence of endo parasites of pig at Mymensingh, Bangladesh. Journal of Agriculture and Veterinary Science, 7, 31–38. 10.6084/M9.FIGSHARE.1226224

[vms3536-bib-0022] Eijck, I., & Borgsteede, F. (2005). A survey of gastrointestinal pig parasites on free‐range, organic and conventional pig farms in the Netherlands. Veterinary Research Communications, 29, 407–414. 10.1007/s11259-005-1201-z 16195935

[vms3536-bib-0023] Ejinaka, O. R. A., & Onyali, I. O. (2020). Parasitic gastrointestinal helminths and protozoa in pigs at Enugu, Nigeria. The Biomedical Diagnostics, 4, 67–74.

[vms3536-bib-0024] Eysker, M., Boerdam, G., Hollanders, W., & Verheijden, J. (1994). The prevalence of *Isospora suis* and *Strongyloides ransomi* in suckling piglets in the Netherlands. Veterinary Quarterly, 16, 203–205. 10.1080/01652176.1994.9694449 7740744

[vms3536-bib-0025] Farzan, A., Parrington, L., Coklin, T., Cook, A., Pintar, K., Pollari, F., Friendship, R., Farber, J., & Dixon, B. (2011). Detection and characterization of *Giardia duodenalis* and *Cryptosporidium* spp. on swine farms in Ontario, Canada. Foodborne Pathogens and Disease, 8, 1207–1213. 10.1089/fpd.2011.0907 21675863

[vms3536-bib-0026] Gatenby, R. M., & Chemjong, P. B. (1992). Reproduction of pigs in the hills of eastern Nepal. Tropical Animal Health and Production, 24, 135–142. 10.1007/BF02359602 1304659

[vms3536-bib-0027] Ghimire, T. R., & Bhattarai, N. (2019). A survey of gastrointestinal parasites of goats in a goat market in Kathmandu. Nepal. Journal of Parasitic Diseases, 43, 686–695. 10.1007/s12639-019-01148-w 31749541 PMC6841857

[vms3536-bib-0028] Giese, W., Dey‐Hazra, A., & Enigk, K. (1973). Enteric loss of plasma‐proteins in *Strongyloides*‐infection of pigs. International Journal for Parasitology, 3, 631–639. 10.1016/0020-7519(73)90088-x 4741639

[vms3536-bib-0029] GoN . (2019). Economic survey 2018/19 (Ministry of Finance , Ed.). Kathmandu: Government of Nepal (GoN), Ministry of Finance. https://www.mof.gov.np/uploads/document/file/compiled%20economic%20Survey%20english%207‐25_20191111101758.pdf

[vms3536-bib-0031] Henry, S. C., & Tokach, L. M. (1995). *Eimeria*‐associated pathology in breeding gilts. Swine Health and Production, 3, 200–201.

[vms3536-bib-0032] Horchner, F., & Dalchow, W. (1972). Experimental *Fasciola hepatica* infection in pigs. Berliner Und Münchener Tierärztliche Wochenschrift, 85, 184–188. 5044569

[vms3536-bib-0033] Ismail, H. A. H. A., Jeon, H.‐K., Yu, Y.‐M., Do, C., & Lee, Y.‐H. (2010). Intestinal parasite infections in pigs and beef cattle in rural areas of Chungcheongnam‐do, Korea. The Korean Journal of Parasitology, 48, 347–349. 10.3347/kjp.2010.48.4.347 21234241 PMC3018588

[vms3536-bib-0034] Jacob, A. S., Busby, E. J., Levy, A. D., Komm, N., & Clark, C. G. (2016). Expanding the *Entamoeba* universe: New hosts yield novel ribosomal lineages. Journal of Eukaryotic Microbiology, 63, 69–78. 10.1111/jeu.12249 26130044

[vms3536-bib-0035] Ji, T., Cao, H.‐X., Wu, R., Cui, L.‐L., Su, G.‐M., Niu, C., Zhang, N., Wang, S.‐K., & Zhou, D.‐H. (2019). Prevalence and genetic identification of three *Entamoeba* Species in pigs in Southeastern China. BioMed Research International, 2019. 10.1155/2019/2824017 PMC6875202 31781607

[vms3536-bib-0036] Johnson, J., Samarasinghe, B., Buddle, R., Armson, A., & Ryan, U. (2008). Molecular identification and prevalence of *Isospora* sp. in pigs in Western Australia using a PCR–RFLP assay. Experimental Parasitology, 120, 191–193. 10.1016/j.exppara.2008.06.005 18601925

[vms3536-bib-0037] Jones, G., Parker, R., & Parke, C. (1985). Coccidia associated with enteritis in grower pigs. Australian Veterinary Journal, 62, 319. 10.1111/j.1751-0813.1985.tb14917.x 4074221

[vms3536-bib-0038] Kagira, J. M., Kanyari, P. N., Githigia, S. M., Maingi, N., Chege Ng'ang'a, J., & Gachohi, J. M. (2012). Risk factors associated with occurrence of nematodes in free range pigs in Busia District, Kenya. Tropical Animal Health and Production, 44, 657–664. 10.1007/s11250-011-9951-9 21833678

[vms3536-bib-0039] Kváč, M., Hanzlíková, D., Sak, B., & Květoňová, D. (2009). Prevalence and age‐related infection of *Cryptosporidium suis*, *C. muris* and *Cryptosporidium* pig genotype II in pigs on a farm complex in the Czech Republic. Veterinary Parasitology, 160, 319–322. 10.1016/j.vetpar.2008.11.007 19091471

[vms3536-bib-0040] Kváč, M., Kestřánová, M., Pinková, M., Květoňová, D., Kalinová, J., Wagnerová, P., Kotková, M., Vítovec, J., Ditrich, O., & Mcevoy, J. (2013). *Cryptosporidium scrofarum* n. sp. (Apicomplexa: Cryptosporidiidae) in domestic pigs (*Sus scrof*a). Veterinary Parasitology, 191, 218–227. 10.1016/j.vetpar.2012.09.005 23021264 PMC3525736

[vms3536-bib-0041] Laber, K. E., Whary, M. T., Bingel, S. A., Goodrich, J. A., Smith, A. C., & Swindle, M. M. (2002). Biology and diseases of swine. Laboratory Animal Medicine, 615–673. 10.1016/B978-012263951-7/50018-1

[vms3536-bib-0042] Lai, M., Zhou, R., Huang, H., & Hu, S. (2011). Prevalence and risk factors associated with intestinal parasites in pigs in Chongqing, China. Research in Veterinary Science, 91, e121–e124. 10.1016/j.rvsc.2011.01.025 21349561

[vms3536-bib-0043] Mas‐Coma, S., Rodriguez, A., Bargues, M., Valero, M., Coello, J., & Angles, R. (1997). Secondary reservoir role of domestic animals other than sheep and cattle in fascioliasis transmission in the Northern Bolivian Altiplano. Research and Reviews in Parasitology, 57, 39–46.

[vms3536-bib-0044] Matsubayashi, M., Kita, T., Narushima, T., Kimata, I., Tani, H., Sasai, K., & Baba, E. (2009). Coprological survey of parasitic infections in pigs and cattle in slaughterhouse in Osaka, Japan. Journal of Veterinary Medical Science, 71, 1079–1083. 10.1292/jvms.71.1079 19721362

[vms3536-bib-0045] Matsubayashi, M., Murakoshi, N., Komatsu, T., Tokoro, M., Haritani, M., & Shibahara, T. (2015). Genetic identification of *Entamoeba polecki* subtype 3 from pigs in Japan and characterisation of its pathogenic role in ulcerative colitis. Infection, Genetics and Evolution, 36, 8–14. 10.1016/j.meegid.2015.08.030 26318541

[vms3536-bib-0046] Matsubayashi, M., Suzuta, F., Terayama, Y., Shimojo, K., Yui, T., Haritani, M., & Shibahara, T. (2014). Ultrastructural characteristics and molecular identification of *Entamoeba suis* isolated from pigs with hemorrhagic colitis: Implications for pathogenicity. Parasitology Research, 113, 3023–3028. 10.1007/s00436-014-3965-y 24894081

[vms3536-bib-0047] Mendoza‐Gómez, M. F., Pulido‐Villamarín, A., Barbosa‐Buitrago, A., & Aranda‐Silva, M. (2015). Presence of gastrointestinal parasites in pigs and humans from four pig farms in Cundinamarca – Colombia. Revista MVZ Córdoba, 26, 5014–5027. 10.21897/rmvz.15

[vms3536-bib-0048] Meyer, C., Joachim, A., & Daugschies, A. (1999). Occurrence of *Isospora suis* in larger piglet production units and on specialized piglet rearing farms. Veterinary Parasitology, 82, 277–284. 10.1016/s0304-4017(99)00027-8 10384903

[vms3536-bib-0049] Midttun, H. L., Acevedo, N., Skallerup, P., Almeida, S., Skovgaard, K., Andresen, L., Skov, S., Caraballo, L., Van Die, I., & Jørgensen, C. B. (2018). *Ascaris suum* infection downregulates inflammatory pathways in the pig intestine in vivo and in human dendritic cells in vitro. The Journal of Infectious Diseases, 217, 310–319. 10.1093/infdis/jix585 29136163

[vms3536-bib-0050] Minetti, C., Taweenan, W., Hogg, R., Featherstone, C., Randle, N., Latham, S., & Wastling, J. (2014). Occurrence and diversity of *Giardia duodenalis* assemblages in livestock in the UK. Transboundary and Emerging Diseases, 61, e60–e67. 10.1111/tbed.12075 23472706 PMC4285228

[vms3536-bib-0051] Morgan, U., Buddle, J., Armson, A., Elliot, A., & Thompson, R. (1999). Molecular and biological characterisation of *Cryptosporidium* in pigs. Australian Veterinary Journal, 77, 44–47. 10.1111/j.1751-0813.1999.tb12428.x 10028394

[vms3536-bib-0052] Murthy, C. K., Ananda, K., Adeppa, J., & Satheesha, M. (2016). Studies on gastrointestinal parasites of pigs in Shimoga region of Karnataka. Journal of Parasitic Diseases, 40, 885–889. 10.1007/s12639-014-0598-0 27605803 PMC4996211

[vms3536-bib-0053] Nansen, P., Andersen, S., Harmer, E., & Riising, H.‐J. (1972). Experimental fascioliasis in the pig. Experimental Parasitology, 31, 247–254. 10.1016/0014-4894(72)90115-4 4622737

[vms3536-bib-0054] Nidup, K., Joshi, D. D., Gongora, J., & Moran, C. (2010). Farming and biodiversity of indigenous pigs in Nepal. Biodiversity, 11, 26–33. 10.1080/14888386.2010.9712661

[vms3536-bib-0055] Nissen, S., Poulsen, I. H., Nejsum, P., Olsen, A., Roepstorff, A., Rubaire‐Akiiki, C., & Thamsborg, S. M. (2010). Prevalence of gastrointestinal nematodes in growing pigs in Kabale District in Uganda. Tropical Animal Health and Production, 43, 567–572. 10.1007/s11250-010-9732-x 21088893

[vms3536-bib-0056] Nonga, H. E., & Paulo, N. (2015). Prevalence and intensity of gastrointestinal parasites in slaughter pigs at Sanawari slaughter slab in Arusha, Tanzania. Livestock Research for Rural Development, 27. http://www.lrrd.org/lrrd27/1/nong27010.htm

[vms3536-bib-0057] Nsoso, S., Mosala, K., Ndebele, R., & Ramabu, S. (2000). The prevalence of internal and external parasites in pigs of different ages and sexes in Southeast District, Botswana. Onderstepoort Journal of Veterinary Research, 67, 217–220. 11131123

[vms3536-bib-0058] Nwafor, I. C., Roberts, H., & Fourie, P. (2019). Prevalence of gastrointestinal helminths and parasites in smallholder pigs reared in the central Free State Province. Onderstepoort Journal of Veterinary Research, 86, 1687. 10.4102/ojvr.v86i1.1687 PMC6495001 31038321

[vms3536-bib-0059] Obonyo, F. O., Maingi, N., Githigia, S. M., & Ng'ang'a, C. J. (2013). Farming practices and risk factors for transmission of helminths of free range pigs in Homabay District, Kenya. Livestock Research for Rural Development, 25. http://www.lrrd.org/lrrd25/3/bon25036.htm

[vms3536-bib-0060] Ondrejková, A., Černek, Ľ., Prokeš, M., Ondrejka, R., Hurníková, Z., & Takáčová, D. (2012). Monitoring of *Ascaris suum* in slaughter pigs during 2000–2009 in Slovakia. Helminthologia, 49, 221–224. 10.2478/s11687-012-0041-y

[vms3536-bib-0061] Pant, K. R. (2017). Pig farming in Nepal is growing step by step. In Pig progress: World of pigs. The Netherlands: Misset Uitgeverij B.V. https://www.pigprogress.net/World‐of‐Pigs1/Articles/2017/9/Pig‐farming‐in‐Nepal‐is‐growing‐step‐by‐step‐177536E/

[vms3536-bib-0062] Patra, G., Al‐Abodi, H. R., Sahara, A., Ghosh, S., Borthakur, S. K., Polley, S., Behera, P., & Deka, A. (2019). Prevalence of parasitic fauna of pigs in North‐Eastern region of India. Biological Rhythm Research, 51(8), 1298–1315. 10.1080/09291016.2019.1573460

[vms3536-bib-0063] Patra, G., Prasad, H., Lalsiamthara, J., Kataria, J., Malsawmkima, D., & Lalrinkima, H. (2013). Lungworm infestation in piglets in different parts of Mizoram, India. Research Journal of Parasitology, 8, 37–44. 10.3923/jp.2013.37.44

[vms3536-bib-0030] Paudel, L.N., Parajuli, D., & Achhami, K. (2014). Present status, trend and potentiality of pig industry in Nepal. In T. B. Gurung, B. S. Shrestha, R. Bates, D. Neupane, T. Paudel, K. Achhami, & N. P. Shrestha (Eds.), Pig and pork industry in Nepal Proceedings of the First National Workshop on Pig & Pork Industry in Nepal held on Dec 10–11, 2013 Kathmandu, Nepal (pp. 8–16). Thematic Area I: Policy and Enabling Environment. Nepal Agricultural Research Council in close collaboration with Department of Livestock Services, SAMARTH/NMDP, CEAPRED, UKaid, Michigan State University. http://elibrary.narc.gov.np/pages/view.php?ref=4068&k=

[vms3536-bib-0064] Petersen, H. H., Jianmin, W., Katakam, K. K., Mejer, H., Thamsborg, S. M., Dalsgaard, A., Olsen, A., & Enemark, H. L. (2015). *Cryptosporidium* and *Giardia* in Danish organic pig farms: Seasonal and age‐related variation in prevalence, infection intensity and species/genotypes. Veterinary Parasitology, 214, 29–39. 10.1016/j.vetpar.2015.09.020 26483166

[vms3536-bib-0065] Ross, J., Dow, C., & Todd, J. (1967). The pathology of *Fasciola hepatica* infection in pigs: A comparison of the infection in pigs and other hosts. British Veterinary Journal, 123, 317–322. 10.1016/s0007-1935(17)39909-8 6065336

[vms3536-bib-0066] Sah, R. (2018). Prevalence of common gastrointestinal nematode parasites in pigs based on different altitudes and seasons in Dhankuta and Sunsari districts of Nepal. Nepalese Journal of Agricultural Sciences, 16, 77–84. https://www.cabi.org/vetmedresource/abstract/20183359766

[vms3536-bib-0067] Salvador, A., Veiga, J. P., Martin, J., Lopez, P., Abelenda, M., & Puertac, M. (1996). The cost of producing a sexual signal: Testosterone increases the susceptibility of male lizards to ectoparasitic infestation. Behavioral Ecology, 7, 145–150. 10.1093/beheco/7.2.145

[vms3536-bib-0068] Sangioni, L. A., De Avila Botton, S., Ramos, F., Cadore, G. C., Monteiro, S. G., Pereira, D. I. B., & Vogel, F. S. F. (2017). *Balantidium coli* in pigs of distinct animal husbandry categories and different hygienic‐sanitary standards in the central region of Rio grande do sul state. Brazil. Acta Scientiae Veterinariae, 45, 6. 10.22456/1679-9216.80041

[vms3536-bib-0069] Sarashina, T., & Taniyama, H. (1986). A case of *Hyostrongylus rubidus* infection in a pig. The Japanese Journal of Veterinary Science, 48, 163–167. 10.1292/jvms1939.48.163 3959373

[vms3536-bib-0070] Schjørring, S., & Koella, J. C. (2003). Sub‐lethal effects of pathogens can lead to the evolution of lower virulence in multiple infections. Proceedings of the Royal Society of London. Series B: Biological Sciences, 270, 189–193. 10.1098/rspb.2002.2233 12590759 PMC1691217

[vms3536-bib-0071] Schuster, F. L., & Ramirez‐Avila, L. (2008). Current world status of *Balantidium coli* . Clinical Microbiology Reviews, 21, 626–638. 10.1128/CMR.00021-08 18854484 PMC2570149

[vms3536-bib-0072] Seguel, M., & Gottdenker, N. (2017). The diversity and impact of hookworm infections in wildlife. International Journal for Parasitology: Parasites and Wildlife, 6, 177–194. 10.1016/j.ijppaw.2017.03.007 28765810 PMC5526439

[vms3536-bib-0073] Serrano, E., & Millán, J. (2014). What is the price of neglecting parasite groups when assessing the cost of co‐infection? Epidemiology & Infection, 142, 1533–1540. 10.1017/S0950268813002100 24040768 PMC9151198

[vms3536-bib-0074] Sharma, D., Singh, N., Singh, H., & Rath, S. (2020). Copro‐prevalence and risk factor assessment of gastrointestinal parasitism in Indian domestic pigs. Helminthologia, 57, 28–36. 10.2478/helm-2020-0011 32063737 PMC6996252

[vms3536-bib-0075] Soulsby, E. J. L. (2012). Helminths, arthropods and protozoa of domesticated animals (7th ed.). Affiliated East‐West Press Private Limited.

[vms3536-bib-0076] Sowemimo, O., Asaolu, S., Adegoke, F., & Ayanniyi, O. (2012). Epidemiological survey of gastrointestinal parasites of pigs in Ibadan, Southwest Nigeria. Journal of Public Health and Epidemiology, 4, 294–298. 10.5897/JPHE12.042

[vms3536-bib-0077] Steenhard, N., Storey, P., Yelifari, L., Pit, D., Nansen, P., & Polderman, A. (2000). The role of pigs as transport hosts of the human helminths *Oesophagostomum bifurcum* and *Necator americanus* . Acta Tropica, 76, 125–130. 10.1016/S0001-706X(00)00077-2 10936571

[vms3536-bib-0078] Tamboura, H., Banga‐Mboko, H., Maes, D., Youssao, I., Traore, A., Bayala, B., & Dembele, M. (2006). Prevalence of common gastrointestinal nematode parasites in scavenging pigs of different ages and sexes in eastern centre province, Burkina Faso. Onderstepoort Journal of Veterinary Research, 73, 53–60. 10.4102/ojvr.v73i1.169 16715878

[vms3536-bib-0079] Tan, T., Low, V., Lee, S., Chandrawathani, P., Premaalatha, B., & Yvonne, A. (2014). Gastro‐intestinal parasitism among two swine populations in Malaysia: Highlighting the zoonotic transmissible protozoan *Balantidium coli* infections. Malaysian Journal of Veterinary Research, 5, 63–68. http://www.dvs.gov.my/dvs/resources/user_14/MJVR_V5N2/MJVR‐V5N2‐web‐p9.pdf

[vms3536-bib-0080] Thapa, S. (2018). Nepal's untapped potential: Pork meat industry [Online]. Biruwa Advisors Pvt. Ltd. Available from http://biruwa.net/2018/01/nepals‐untapped‐potentialpork‐meat‐industry/?fbclid=IwAR0nvLRAfjCCGMgLmO‐nfuW3l35FFQ9DlpB033xh8E0W4dMf2SnduH8Ho8g. Accessed on September 13, 2020

[vms3536-bib-0081] Uysal, H. K., Boral, O., Metiner, K., & Ilgaz, A. (2009). Investigation of intestinal parasites in pig feces that are also human pathogens. Turkish Journal of Parasitology, 33, 218–221. 19851968

[vms3536-bib-0082] Vaumourin, E., Vourc'h, G., Gasqui, P., & Vayssier‐Taussat, M. (2015). The importance of multiparasitism: Examining the consequences of co‐infections for human and animal health. Parasites & Vectors, 8, 1–13. 10.1186/s13071-015-1167-9 26482351 PMC4617890

[vms3536-bib-0083] Widisuputri, N. K. A., Suwanti, L. T., & Plumeriastuti, H. (2020). A Survey for zoonotic and other gastrointestinal parasites in pig in Bali Province, Indonesia. Indonesian Journal of Tropical and Infectious Disease, 8, 55–66. 10.20473/ijtid.v8i1.10393

[vms3536-bib-0084] Worliczek, H. L., Buggelsheim, M., Saalmüller, A., & Joachim, A. (2007). Porcine isosporosis: Infection dynamics, pathophysiology and immunology of experimental infections. Viennese Clinical Weekly, 119, 33–39. 10.1007/s00508-007-0859-3 17987356

[vms3536-bib-0085] Xiao, L., Herd, R., & Bowman, G. (1994). Prevalence of *Cryptosporidium* and *Giardia* infections on two Ohio pig farms with different management systems. Veterinary Parasitology, 52, 331–336. 10.1016/0304-4017(94)90124-4 8073616

[vms3536-bib-0086] Xiao, L., Moore, J. E., Ukoh, U., Gatei, W., Lowery, C. J., Murphy, T. M., Dooley, J. S., Millar, B. C., Rooney, P. J., & Rao, J. R. (2006). Prevalence and identity of *Cryptosporidium* spp. in pig slurry. Applied and Environmental Microbiology, 72, 4461–4463. 10.1128/AEM.00370-06 16751569 PMC1489634

[vms3536-bib-0087] Zhang, S.‐X., Zhou, Y.‐M., Xu, W., Tian, L.‐G., Chen, J.‐X., Chen, S.‐H., Dang, Z.‐S., Gu, W.‐P., Yin, J.‐W., & Serrano, E. (2016). Impact of co‐infections with enteric pathogens on children suffering from acute diarrhea in southwest China. Infectious Diseases of Poverty, 5, 64. 10.1186/s40249-016-0157-2 27349521 PMC4922062

