# Supplementary material for: Gastrointestinal parasites of indigenous pigs (Sus domesticus) in south‐central Nepal
Source: Vet Med Sci. 2021 May 22;7(5):1820–30. doi: 10.1002/vms3.536 (PMC8464252; doi:10.1002/vms3.536)
Supplement: Supplementary file 1 — Table S1 [file VMS3-7-1820-s002.docx]

| ***Eimeria* species** | **Prevalence (%)** | **Oocyst characteristics** | | | **Sporocyst characteristics** | | |
| --- | --- | --- | --- | --- | --- | --- | --- |
|  |  | **Length range (average, SEM) (µm) x Width range (average, SEM) (µm*)*** | **Shape index (l/b*)*** | **Shape of oocyst** | **Length (µm) x Width (µm)** | **Shape index (l/b)** | **Shape of sporocyst** |
| ***E. debliecki*** | 32 | 22–28 (24.7,0.2) x14–20 (17.4,0.2), n=60 | 1.4 | Ovoid | 9–13 (11.2,0.3) x5–8 (6.1,0.1), n=33 | 1.8 | Ellipsoidal |
| ***E. neodebliecki*** | 40 | 18–21 (20,0.1) x13–17 (15,0.1), n=70 | 1.3 | Ellipsoidal | 8–12 (10.2,0.1) x4–7 (5.4,0.1), n=59 | 1.9 | Ellipsoidal |
| ***E. perminuta*** | 25 | 14–18 (16.0,0.2) x10–15 (12.9,0.2), n=45 | 1.2 | Ovoid and Spherical | 5–8 (6.4,0.2) x4–6 (5.2,0.2), n=14 | 1.2 | Ovoid |
| ***E. polita*** | 16 | 24–30 (26.8,0.4) x19–22 (20.1,0.2), n=20 | 1.3 | Ellipsoidal and occasionally Broad to Oval | 11–14 (12.8,0.3) x6–8 (6.8,0.2), n= 12 | 1.9 | Ellipsoidal |
| ***E. porci*** | 24 | 20–24 (22.2,0.2) x14–17 (15.7,0.2), n=40 | 1.4 | Ovoid | 7–10 (8.5,0.3) x5–7 (6.1,0.2), n=15 | 1.4 | Spherical to Ovoid |
| ***E. scabra*** | 1 | 29x20, n=1 | 1.5 | Ellipsoidal to Ovoid | 13x6, n=1 | 2.2 | Elongated and Ellipsoidal |
| ***E. suis*** | 26 | 17–21 (18.7,0.1) x12–16 (14.6,0.1), n=52 | 1.28 | Ovoid | 7–10 (8.3,0.1) x4–7 (5.3,0.2), n=27 | 1.6 | Ellipsoidal |

**SUPPLEMENTARY TABLE S1 Oocyst and Sporocyst characteristics of *Eimeria* species and their prevalence (%).** SEM: Standard Error from Mean. n= number of specimen examined.
