# Supplementary material for: Gastrointestinal parasites of indigenous pigs (Sus domesticus) in south‐central Nepal
Source: Vet Med Sci. 2021 May 22;7(5):1820–30. doi: 10.1002/vms3.536 (PMC8464252; doi:10.1002/vms3.536)
Supplement: Supplementary file 2 — Table S2 [file VMS3-7-1820-s001.docx]

**Supporting Information Table S2.** Pattern of infection with its prevalence (%) in different groups of pigs.

| **Group of Pigs** | **Pattern of infection** | **Prevalence (%)** |
| --- | --- | --- |
| **Sucklings and Weaners (n= 30)** | **Single** | |
|  | *Entamoeba* | 3 |
|  | *Eimeria* | 2 |
|  | *Strongyloides* | 1 |
|  | **Double** | |
|  | *Entamoeba* + *Eimeria* | 1 |
|  | *Entamoeba* + *Cystoisospora* | 1 |
|  | *E. coli + Eimeria* | 1 |
|  | *Eimeria + Strongyloides* | 1 |
|  | **Triplet** | |
|  | *Entamoeba + Eimeria + Cystoisospora* | 1 |
|  | *Entamoeba + Cystoisospora + Strongyloides* | 1 |
|  | *Entamoeba + Cystoisospora+* Ascarid | 1 |
|  | *Entamoeba + Eimeria + Cryptosporidium* | 1 |
|  | *Entamoeba + Cystoisospora + Strongyloides* | 1 |
|  | *Eimeria + B. coli + Strongyloides* | 1 |
|  | *Entamoeba + Eimeria + B. coli* | 1 |
|  | *Entamoeba + Giardia + Cystoisospora* | 1 |
|  | *Entamoeba + Eimeria + Trichuris* | 1 |
|  | *Entamoeba + Cryptosporidium + Trichuris* | 1 |
|  | **Quadruplet** | |
|  | *Entamoeba + Giardia + Cryptosporidium + Trichuris* | 1 |
|  | *Entamoeba + Eimeria+ Strongyloides +* Hookworm | 1 |
|  | *Eimeria + Giardia + B. coli + Cryptosporidium* | 1 |
|  | *Entamoeba + Cryptosporidium + Cystoisospora +* Ascarid | 1 |
|  | *Entamoeba + Eimeria + Strongyloides +* Hookworm | 1 |
|  | *Eimeria + Giardia +* Hookworm *+ Trichuris* | 1 |
|  | *Entamoeba + B. coli + Trichuris +* Ascarid | 1 |
| **Growers (n=30)** | **Double** | |
|  | *Entamoeba +* Ascarid | 1 |
|  | *Eimeria + B. coli* | 1 |
|  | Ascarid + Strongyle | 1 |
|  | **Triplet** | |
|  | *Entamoeba + Giardia + Trichuris* | 1 |
|  | *Entamoeba +* Ascarid *+* Strongyle | 2 |
|  | *Entamoeba + Eimeria + Trichuris* | 1 |
|  | *E. coli +* Strongyle *+ Strongyloides* | 1 |
|  | *Eimeria + B. coli +* Ascarid | 1 |
|  | **Quadruplet** | |
|  | *Entamoeba + Eimeria +* Ascarid *+ Fasciola* | 1 |
|  | *Isospora +* Ascarid *+ Trichuris +* Hookworm | 1 |
|  | *Entamoeba + E. coli +* Ascarid *+* Strongyle | 1 |
|  | *Entamoeba + Eimeria + Cryptosporidium +* Ascarid | 1 |
|  | *Entamoeba + Eimeria + Cystoisospora + Trichuris* | 1 |
|  | *Entamoeba + Eimeria + Strongyloides + B. coli* | 1 |
|  | *Entamoeba + B. coli + Cystoisospora+ Strongyle* | 1 |
|  | *B. coli +* Ascarid *+ Trichuris +* Strongyle | 1 |
|  | *Eimeria + B. coli + Fasciola + Trichuris* | 1 |
|  | *Entamoeba +* Strongyle *+ Strongyloides + Fasciola* | 1 |
|  | *Eimeria + Cryptosporidium +* Ascarid *+ Trichuris* | 1 |
|  | *Eimeria + B. coli +* Hookworm *+ Strongyloides* | 1 |
|  | *Entamoeba + Eimeria + E. coli + Trichuris* | 1 |
|  | *Entamoeba + Eimeria +* Strongyle *+ Fasciola* | 1 |
|  | **Pentuplet** | |
|  | *Entamoeba + E. coli + Eimeria +* Ascarid + Hookworm | 1 |
|  | *Entamoeba + Cystoisospora + Trichuris +* Strongyle *+ Fasciola* | 1 |
|  | *Eimeria + B. coli + E. coli +* Hookworm + Ascarid | 1 |
|  | **Hexuplet** | |
|  | *Entamoeba + Iodamoeba +* Ascarid + Strongyle *+ Strongyloides + Fasciola* | 1 |
|  | *Entamoeba + Eimeria + Cryptosporidium +* Ascarid *+ Trichuris +* Strongyle | 1 |
|  | *Entamoeba + B. coli + Cystoisospora +* Ascarid *+ Trichuris +* Strongyle | 1 |
|  | *Entamoeba + Eimeria + Cystoisospora* *+ Strongyloides +* Strongyle *+ Trichuris* | 1 |
| **Adults (N=40)** | **Double** | |
|  | *Entamoeba +* Ascarid | 1 |
|  | *B. coli +* Ascarid | 1 |
|  | **Triple** | |
|  | *Eimeria + E. coli + B. coli* | 1 |
|  | *Entamoeba + Iodamoeba +* Strongyle | 1 |
|  | *Eimeria + E. coli + Trichuris* | 1 |
|  | **Quadruplet** | |
|  | *E. coli + B. coli +* Strongyle + Ascarid | 1 |
|  | *Entamoeba + Iodamoeba +* Ascarid *+ Strongyloides* | 1 |
|  | *Eimeria +* Ascarid + Strongyle *+ Trichuris* | 1 |
|  | *Entamoeba + B. coli +* Ascarid + Strongyle | 1 |
|  | *Entamoeba + Iodamoeba +* Ascarid *+ Trichuris* | 1 |
|  | *Entamoeba + Cystoisospora +* Ascarid + Strongyle | 1 |
|  | *Entamoeba +* Ascarid *+ Fasciola + Trichuris* | 1 |
|  | *Entamoeba + B. coli +* Hookworm + Strongyle | 1 |
|  | *Entamoeba + Giardia +* Ascarid + Hookworm | 1 |
|  | **Pentuplet** | |
|  | *Entamoeba + Iodamoeba + Eimeria + Strongyloides +* Hookworm | 1 |
|  | *Entamoeba + Eimeria +* Ascarid + Strongyle *+ Strongyloides* | 1 |
|  | *Entamoeba + B. coli +* Ascarid *+ Strongyloides +* Hookworm | 1 |
|  | *Entamoeba + Cryptosporidium +* Ascarid *+ Strongyloides +* Hookworm | 1 |
|  | *Entamoeba + B. coli +* Ascarid + Strongyle + Hookworm | 1 |
|  | *Entamoeba + Cystoisospora +* Ascarid + Strongyle + Hookworm | 1 |
|  | *Entamoeba + B. coli +* Ascarid *+ Strongyloides + Fasciola* | 1 |
|  | *B. coli +* Hookworm + Strongyle *+ Strongyloides + Fasciola* | 1 |
|  | *Entamoeba + Eimeria + Giardia + Cystoisospora + Iodamoeba* | 1 |
|  | *Entamoeba + Eimeria +* Ascarid + Strongyle *+ Fasciola* | 1 |
|  | *Entamoeba + Eimeria +* Ascarid *+ Trichuris +* Hookworm | 1 |
|  | *Entamoeba + Eimeria +* Ascarid + Hookworm *+ Strongyloides* | 1 |
|  | **Hexuplet** | |
|  | *Eimeria + Cystoisospora +* Ascarid + Strongyle + Hookworm *+ Trichuris* | 1 |
|  | *Eimeria + E. coli + B. coli +* Ascarid + Strongyle *+ Trichuris* | 1 |
|  | *Entamoeba + Cystoisospora + B. coli +* Ascarid + Strongyle *+ Trichuris* | 1 |
|  | *E. coli + B. coli + Cystoisospora +* Ascarid *+ Strongyloides + Trichuris* | 1 |
|  | **Septuplet** | |
|  | *Entamoeba + Iodamoeba + Cryptosporidium + B. coli +* Ascarid *+* Strongyle *+ Trichuris* | 1 |
|  | *Eimeria + B. coli +* Ascarid + Strongyle + Hookworm *+ Trichuris + Strongyloides* | 1 |
|  | *Entamoeba + Eimeria + Cystoisospora + B. coli +* Ascarid + Strongyle *+ Trichuris* | 1 |
|  | *Entamoeba + Eimeria + Cystoisospora +* Hookworm *+ Trichuris +* Strongyle *+ Fasciola* | 1 |
