# Supplementary material for: Gastrointestinal parasites of indigenous pigs (Sus domesticus) in south‐central Nepal
Source: Vet Med Sci. 2021 May 22;7(5):1820–30. doi: 10.1002/vms3.536 (PMC8464252; doi:10.1002/vms3.536)
Supplement: Supplementary file 3 — Table S3 [file VMS3-7-1820-s004.docx]

**Supporting Information Table S3** ***Cystoisospora* occurrence concurrent with *Eimeria***.

| **Patterns of infection** | **Suckling and weaners (n=30)** | **Growers**  **(n=30)** | **Adults**  **(n=40)** |
| --- | --- | --- | --- |
| Triplet | 1 (3.3%) | 0 | 0 |
| Quadruplet | 0 | 1 (3.3%) | 0 |
| Pentuplet | 0 | 0 | 1 (2.5%) |
| Hexuplet | 0 | 1(3.3%) | 1 (2.5%) |
| Septuplet | 0 | 0 | 2 (5%) |
| **Overall** | **1 (3.3%)** | **2 (6.7%)** | **4 (10%)** |
