# Supplementary material for: Gastrointestinal parasites of indigenous pigs (Sus domesticus) in south‐central Nepal
Source: Vet Med Sci. 2021 May 22;7(5):1820–30. doi: 10.1002/vms3.536 (PMC8464252; doi:10.1002/vms3.536)
Supplement: Supplementary file 4 — Table S4 [file VMS3-7-1820-s003.docx]

**Supporting Information Table S4 OPG/EPG in the fecal samples with respect to age of the pigs.**

| **Parasites** | **Average range of OPG/EPG** | | |
| --- | --- | --- | --- |
|  | **Suckling and Weaner** | **Growers** | **Adults** |
| ***Eimeria* spp.** | 400–6200 | 500–5800 | 400–4200 |
| ***Cystoisospora* sp.** | 100–1600 | 100–2400 | 100–1200 |
| **Ascarid** | 100–2400 | 100–6500 | 200–7800 |
| **Strongyle** | 0 | 100–4200 | 200–4000 |
| ***Trichuris* spp.** | 100–800 | 100–1100 | 100–1500 |
| ***Strongyloides* sp.** | 100–700 | 100–1400 | 100–2200 |
| **Hookworm** | 100–500 | 100–1400 | 100–1600 |
